# Protective role of sodium propionate against glycerol or fractionated doses of gamma rays-induced acute kidney injury via ATF5-induced mitophagy in rats

**DOI:** 10.1038/s41598-026-46553-3

**Published:** 2026-04-12

**Authors:** Mahmoud E. Habieb, Maha M. Ali, Amira Abd-ElRaouf, Fatma Y. Abdou

**Affiliations:** Drug Radiation Research Department, National Center for Radiation Research and Technology (NCRRT), Egyptian Atomic Energy Authority (EAEA), Cairo, Egypt

**Keywords:** Acute kidney injury, Gamma radiation, Sodium propionate, Mitophagy, Glycerol, Rats, Biochemistry, Drug discovery, Medical research, Nephrology, Physiology

## Abstract

Acute kidney injury (AKI), whether induced by nephrotoxins like glycerol or by gamma radiation, is characterized by severe oxidative stress and subsequent mitochondrial dysfunction. We investigated the protective mechanism of sodium propionate (SP) against AKI in a rat model. Six experimental groups were established: (I) control rats were given saline; (II) rats were administered SP (37.5 mg/kg, p.o.) for two weeks; (III) rats were given an intramuscular injection of glycerol 10 mL/kg body weight; (IV) rats were given glycerol followed by SP treatment for two weeks; (V) rats were exposed to fractionated gamma-radiation (8 Gy; delivered as 2 Gy x 4 times); and (VI) γ-irradiated rats were treated with SP for two weeks. In comparison to AKI rats, SP treatment significantly preserved renal function, reduced serum urea and creatinine, and improved histopathological features. Biochemically, SP reduced lipid peroxidation and protein oxidation (malondialdehyde MDA, protein carbonyl PC, and lipofuscin) while restoring antioxidant defenses as reduced glutathione (GSH) and methionine sulfoxide reductase A (MSRA). SP restored mitophagy flux by increasing microtubule-associated protein light chain 3 (LC3II/LC3I) ratio and PTEN-induced putative kinase 1 (PINK-1) levels, promoting p62 clearance, and downregulating the mitochondrial stress marker, activating transcription factor 5 (ATF5), relative to the untreated AKI groups. These findings demonstrate that SP confers protection against AKI by attenuating oxidative stress and re-establishing mitochondrial quality control through re-establishment of autophagic flux. Hence, SP represents a promising candidate for therapeutic intervention in nephrotoxin- and γ-radiation-induced renal injury.

**Supplementary Information:**

The online version contains supplementary material available at 10.1038/s41598-026-46553-3.

## Introduction

Acute kidney injury (AKI) is a serious medical illness characterized by abrupt and dramatic decreases in renal function^1^. It is detected following the development of progressive azotemia, which leads to a high death rate^2^. Ischemia damage and nephrotoxic exposures are two of the many etiologies of AKI, a complicated and potentially fatal condition^3^. Inflammation is a key component of AKI^4^. It induces the extension phase of the damage, which may be related to resistance to vasodilator treatment^5^. It is hypothesized that targeting the extension phase is a potential area of treatment with the most effect^6^. The primary idea behind renal tissue damage seems to be the impairment of the renal outer medulla’s highly metabolically active nephron segments, which can cause the transition from transient hypoxia to intrinsic renal failure^7^.

The leading cause of AKI is acute tubular necrosis which can be caused by ischemia, exogenous nephrotoxic chemicals, or endogenous nephrotoxic damage from rhabdomyolysis and hemolysis^8,9^. There are no effective medications to prevent or cure AKI, and the mortality rate is still startlingly high even though most patients who survive can reverse the loss of renal function^10,11^. The most popular experimental paradigm for studying this injury is glycerol-induced AKI^12^ and is distinguished by severe cortical acute tubular necrosis and inflammatory cell infiltration^13^.

Glycerol-induced AKI in rodents is caused by renal ischemia and myoglobin nephrotoxicity^14^. In glycerol-induced AKI, the redox cycle and myoglobin heme promote oxidative stress and lipid peroxidation in the proximal tubular cells. This triggers a cascade of mediators, including cytokines and chemokines, resulting in leukocyte activation and tubular necrosis in the cortical region^15^. Myoglobin-induced kidney toxicity promotes oxidative stress, inflammation, endothelial dysfunction, vasoconstriction, and apoptosis, all of which contribute significantly to acute renal injury^16,17^. However, the particular molecular mechanism of glycerol-induced nephrotoxicity is unknown^18^.

Glycerol-induced ischemia shock to the kidneys may cause the phospholipid membranes in the cellular to be disturbed^19^. This could set off a series of biochemical reactions that cause irreversible cell damage, most likely as a result of increased production of free radicals derived from oxygen and tissue phospholipases^20^. It has been shown that glycerol injection can cause acute renal failure by rhabdomyolysis in a variety of in vivo and ex vivo experimental settings using laboratory animals. Muscle cell membranes break down when glycerol is present, releasing proteins that contain iron into the extracellular environment^21^. Damage to the proximal and distal convoluted tubules in the renal cortex caused by the buildup of protein-containing cells in the renal tubules and the constriction of renal blood vessels in response to adenosine could result in acute renal failure^22^.

Glycerol is a chemical molecule that is hygroscopic and water soluble due to its three hydrophilic alcohol hydroxyl groups^23^. Triacylglycerols, phospholipids, and glucose (via gluconeogenesis), are all synthesized from glycerol, which is a key component of the structure of many lipids^24^. Depending on the physiological circumstances, exogenous glycerol absorbed from dietary lipids is released into the bloodstream after digestion and may enter the gluconeogenesis or glycolysis pathways^25^. Glycerol kinase (GK), which is predominantly found in the liver and kidney, catalyzes the phosphorylation of glycerol in glycerol 3-phosphate (G3P). Glycerol 3-phosphate dehydrogenase (GPDH), an enzyme found in the heart, kidney, liver, and skeletal and smooth muscles, catalyzes the reversible oxidation of G3P^26^. Glycerol induces myoglobinuria and rhabdomyolysis in this model, which leads to the development of kidney damage. It was proposed that the primary mechanisms include direct myoglobin-induced cytotoxicity, renal vasoconstriction, and tubular blockage. Numerous investigations have demonstrated the part oxidative stress plays in the pathophysiology, which is believed to include vascular congestion, cast formation, and oxidative stress^27,28^.

Radiotherapy dose fractionation was used to increase the therapeutic ratio, reduce radiation’s deleterious effects on healthy cells, and aid in their recovery or repair between treatments^29^. Even when using radiation dose fractionation, nephropathy is unavoidable^30,31^. Radiation nephropathy (RN) is known to cause renal endothelial dysfunction and altered hemodynamics. Furthermore, the increasing loss of kidney function is accompanied by tubular cell loss and increased interstitial scarring^32^. RN represents another established AKI model.

Radiation-induced damage is primarily produced by the production of reactive oxygen species (ROS). Proteins, lipids, and DNA are oxidized as a result of the imbalance between pro-oxidant and antioxidant molecules within the cell, eventually leading to death. Apoptosis has also been found to be the mechanism of renal tubular cell death in radiation nephropathy^33^. Cellular senescence, oxidative stress, inflammation, and DNA damage-mediated cell death are some of the main mechanisms of RN that have been described. It is impossible to totally eliminate radiation-induced kidney damages, even with dose-fractionation efforts. An effective substitute in these situations would be the proactive administration of radioprotectants prior to irradiation^34,35^.

The gut microbiota produces SCFAs, primarily acetate, propionate, and butyrate, by fermenting complex carbohydrates^36^. Propionate and butyrate can inhibit histone deacetylases (HDACs), altering intracellular acetylation patterns and, consequently, gene expression patterns^37^. Anaerobic bacteria in the stomach break down dietary fibers to create SP, a short-chain fatty acid with anti-inflammatory and anti-apoptotic properties via blocking pathways such as NF-κB^38^. However, most studies focus on butyrate; however, some studies have been undertaken on other SCFAs, such as propionate^39^.

Mitophagy is a critical mitochondrial quality control mechanism^40^. When PINK-1 proteolysis is inhibited by damaged mitochondria, PINK-1 collects in the injured mitochondria, and the aggregated PINK-1 then recruits Parkin^41^. The ubiquitination of mitochondrial substrates by Parkin is linked to the occurrence of mitophagy^42^. In response to mitochondrial stress, ATF5 migrates from the cytoplasm to the nucleus, where it starts the mitochondrial unfolded protein response (UPR^mt^) gene cluster. This results in the degradation of misfolded proteins, indicating that UPR^mt^ is activated^43,44^. In tubule cells, oxidative stress and apoptosis are significantly influenced by ATF5. Excessive ATF5 activation may be the mechanism, offering a new therapeutic target to reduce tubular damage^45,46^.

As is widely known, sodium propionate has been demonstrated to regulate oxidative stress and inflammation, two factors that are crucial in kidney disorders^47^. Its precise function in kidney cell mitophagy is still unclear, though. To our knowledge, this is the first study to evaluate SP in both glycerol- or fractionated γ-radiation-induced AKI. Specifically, we investigate whether SP protects renal tissue by modulating oxidative stress and restoring mitophagy via ATF5 regulation. This study makes unique contributions by employing dual injury models, simultaneously evaluating SP in both glycerol-induced AKI and radiation nephropathy to enhance translational relevance. In addition, it identifies ATF5-mediated mitophagy as a critical mechanism in AKI, moving beyond conventional markers such as LC3, PINK-1, and p62, and highlighting ATF5 as a novel regulator of mitophagy that has not been extensively investigated in SCFA research.

## Materials and methods

### Chemicals and reagents

Glycerol and sodium propionate (> 99% purity) were procured from Sigma Chemical Co. (St. Louis, MO). All additional chemicals and kits for biochemical testing were of excellent analytical grade.

### Animals

Adult female albino rats weighting between (180–210 g). The rats were housed in a controlled setting with a normal 12-hour light/dark cycle, a constant temperature of 25 °C, and unfettered access to food, water, and a standard diet of rodent chow. Before the testing began, the animals were acclimatized to the National Center for Radiation Research and Technology’s animal facilities for at least one week. The Animal Care Committee of the National Center for Radiation Research and Technology (NCRRT), which is affiliated with the Egyptian Atomic Energy Authority (EAEA) in Cairo, Egypt, approved the ethical criteria for using animals in research (Permit Number: 46 A/24).

### Irradiation to animals

Animals were treated to 8 Gy fractionated doses, 2 Gy x 4 times every 3 days, at a dose rate of 0.354 Gy/min, utilizing a Cs^137^ irradiation unit at NCRRT, EAEA, Cairo, Egypt. The animals were housed in cages with sufficient ventilation and placed in a chamber connected to the irradiation apparatus. The animals were placed in a field of roughly 25 × 25 cm^2^, 70 cm away from the source.

### Experimental design

**Fig. 1:**
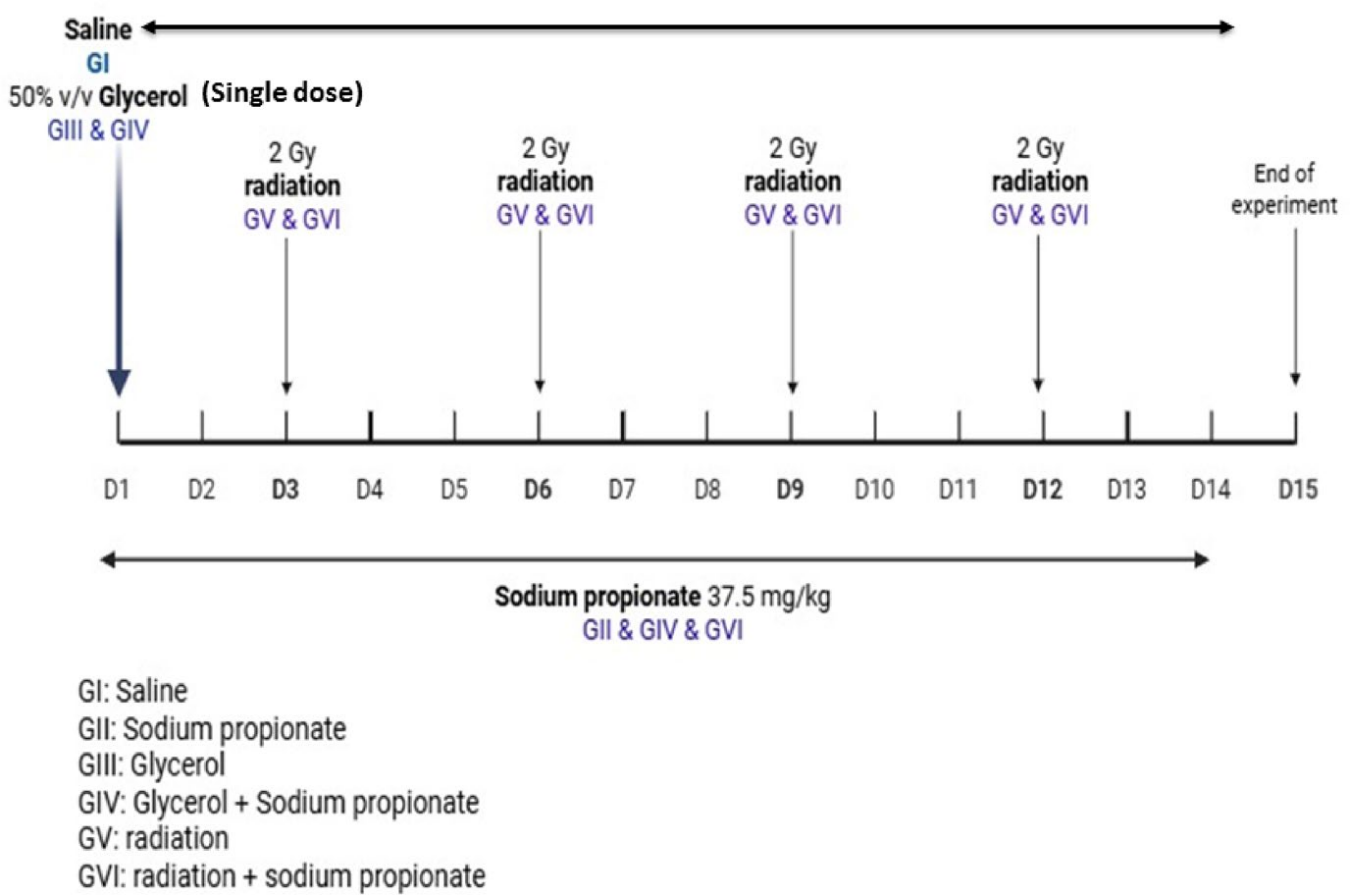
Experimental design.

The rats were randomly distributed into six groups of eight, as follows: Group I: rats were given normal saline for two weeks and acted as negative controls. Group II: rats were given SP (37.5 mg/kg b.w, oral gavage for 14 days)^48^. Group III rats were injected in a single dose intramuscularly (i.m.) with 10 mL/kg b.wt. 50%, v/v Glycerol in a sterile saline (positive control)^10,49^.Group IV: rats were injected by a single i.m. injection of glycerol as in group III followed by SP treatment (37.5 mg/kg b.w.) as in group II for two weeks. Group V: rats were exposed to gamma radiation (8 Gy fractionated doses, 2 Gy х 4 times every 3 day) which serves as the positive control (injury model) for Group VI (IRD + SP). Group VI: rats were exposed to γ-radiation as in group V, then SP (37.5 mg/kg b.w. orally for14 days) during fractionated γ-irradiation and continued daily until the end of exposure period (Fig. 1).

The euthanasia of animals was carried out in compliance with the American Veterinary Medical Association (AVMA) Guidelines for the Euthanasia of Animals (2020)^50^. Twenty-four hours following the last treatment dose, rats were put under anesthesia by injecting thiopental 50 mg/kg intraperitoneally (i.p.)^51^. Blood samples were collected via cardiac puncture to separate the serum.

### Tissue sampling

The kidney tissue was cleaned with ice-cold isotonic saline before being extracted and divided into three parts. First, the first part was weighed and homogenized (10% w/v) in ice-cold phosphate buffer (0.05 M, pH 7.4) before being centrifuged at 3000 rpm for 5 min at 4 °C. The resulting supernatant was stored at −80 °C for later investigation of oxidative stress-related parameters. The second portion was stored at −80 °C for western blotting and biochemical ELISA tests. The third part was stored in 10% neutral formalin to allow for histological and immunohistochemical analysis.

### Biochemical assays

#### Assessment of renal function

Renal function was assessed by monitoring serum urea and creatinine levels using colorimetric commercial kits (Thermo Fisher Scientific Inc., USA) as directed.

#### Oxidative stress biomarkers

Lipid peroxidation was measured according to the method of Yoshioka^52^ involves determining the concentration of malondialdehyde (MDA), a measure of thiobarbituric acid reactive compounds (TBARS). In comparison to the standard n-butanol, the results were represented as nmol of MDA/g wet tissue. GSH (reduced glutathione) was tested using the chemical technique^53^. The results were expressed as mmol of GSH/g wet tissue.

#### ELISA analysis of renal protein carbonyl (PC), methionine sulfoxide reductase A (MSRA), Lipofuscin, PINK-1 and ATF 5

Protein carbonyl, MSRA, lipofuscin, PINK-1 and ATF 5 levels were detected in kidney tissue homogenates using rat Protein carbonyl ELISA Kit (MyBiosource; Cat.no.# MBS2600784), rat methionine sulfoxide reductase A ELISA Kit (MyBiosource; Cat.no.# MBS9319207), rat Lipofuscin ELISA kit (MyBiosource; Cat.no.# MBS3809036), rat Serine/Threonine-Protein Kinase PINK-1, Mitochondrial (PINK-1) ELISA Kit (MyBiosource; Cat.no.# MBS9343426**)**, rat ATF5 ELISA kit (MyBiosource; Cat.no.# MBS9518955). The kits were used according to the manufacturers’ instructions. Using a plate reader, the optical density is determined spectrophotometrically at a wavelength of 450 nm was used for all ELISA-based parameters in this subsection (Protein carbonyl, MSRA, Lipofuscin, PINK-1, and ATF5), as specified by the manufacturers protocols for the respective HRP-labelled detection kits (BMG Labtech, FLU Ostar Omega, Germany).

#### Protein extraction and western blot analysis of LC3II/LC3I ratio

The ReadyPrepTM protein extraction kit (total protein) from Bio-Rad Inc (Cat. no. #163–2086) was used in accordance with the manufacturer’s instructions. Biobasic Inc. (Markham, Ontario, L3R 8T4 Canada) offers the Bradford Protein Assay Kit (SK3041) for quantitative protein analysis. A Bradford assay was used to quantify the protein concentration in each sample, following the manufacturer’s instructions. The samples were separated on a polyacrylamide gel, known as SDS-PAGE (Sodium Dodecyl Sulfate Polyacrylamide Gel Electrophoresis), a common technique for separating proteins based on molecular weight. The SDS-PAGE TGX Stain-Free FastCast was prepared in accordance with the manufacturer’s instructions. Filter paper, PVDF membrane, gel, and filter paper were stacked in a transfer sandwich, bottom to top. The membrane was blocked for one hour at room temperature with tris-buffered saline containing 3% bovine serum albumin (BSA) and Tween 20 (TBST). The primary antibody (Invitrogen; Cat. no. # L10382) was diluted with TBST per the manufacturer’s instructions. The target protein was blotted and incubated in each primary antibody solution at 4 °C overnight. To ensure accurate comparison and loading control, LC3 and β-actin were sequentially detected on the same PVDF membrane by stripping and reprobing, as indicated in the respective figure legend. The target proteins’ band intensities were compared to the control sample, β-actin (a housekeeping protein) using image analysis software on the ChemiDoc MP imager.

### Histopathological examination of kidney tissues

Formalin fixed kidney tissue specimens were processed in ascending grades of ethyl alcohol (80%, 90% and 100%), cleared in xylene and embedded in paraffin wax until the time of diagnostic application. Sections were cut at 5 μm thickness, picked up on glass slides, dried, deparaffinized with xylene and rehydrated with descending grades of ethyl alcohol, washed and stained with H&E according to *Bancroft and Gamble*^54^. The stained sections were dehydrated in absolute ethyl alcohol, cleared in xylene and mounted with Mount-Quick liquid cover glass medium to the surface of the slides. Five multiple fields at 20x magnification of kidney tissue sections were examined and captured under a light digital microscope (Olympus xc30, Tokyo, Japan). Each sample was examined by the same pathologist in a blinded study. Morphological changes were scored from 0 to 4 to assess the degree of renal damage as described by *Jablonski et al.*^55^, **(**Table 1**)**.

**Table 1:** Jablonski scores for the assessment of renal damage.

Score	Vacuolization	Cortical damage	Necrosis
0	None	None	None
1	Minimal	10%	Individual cell necrosis
2	Mild	20%	30%
3	Moderate	30%	60%
4	Severe	> 30%	> 60%

###  Immunohistochemistry P62 expression

For immunological staining, 5 μm slices were cut from pre-formed paraffin tissue blocks and placed on positively charged slides. The heat-induced epitope retrieval stage was carried out with Tris-EDTA buffer (10 mM Tris base, 1 mM EDTA solution, 0.05% Tween 20, pH 9.0), followed by endogenous peroxidase inhibition with hydrogen peroxide. Tissue sections were then incubated with primary anti-P62 (Proteintech; Cat. no. KHC0058) at 1:100 dilutions for an hour at room temperature. For color development, the manufacturer recommended using a supplementary HRP-labelled detection kit (Bio SB, USA). Negative control slides were created by skipping the primary antibody incubation step. Quantitative analysis was performed using Image J software. Integrated optical density (IOD) was measured across five non-overlapping high power fields (HPFs) per slide, and positive staining was expressed as percentage area.

### Statistical analysis

Prior to data analysis, data normality was tested using Kolmogorov-Smirnov tests, while the homogeneity of variances was done using Bartlett test. The parametric data of more than two groups was analyzed using one-way ANOVA, followed by Tukey’s multiple-comparisons post-hoc test, findings were reported as mean ± SE. The non- parametric histology scores were assessed using Kruskal-Wallis nonparametric one-way ANOVA, followed by Dunn’s post-hoc test, and the results were presented as medians (interquartile range). Sample size was calculated using G power software (version 3.1) with given α error = 0.05 and given Power = 0.95, while effect size was calculated using Cohen’s d equation. The statistical analyses were carried out with GraphPad Prism (version 8.4.3). The significance level for each statistical test was *p* < 0.05.

## Results

### Protective role of SP on kidney function tests against glycerol or fractionated dose of gamma-rays-induced AKI in rats

**Fig. 2:**
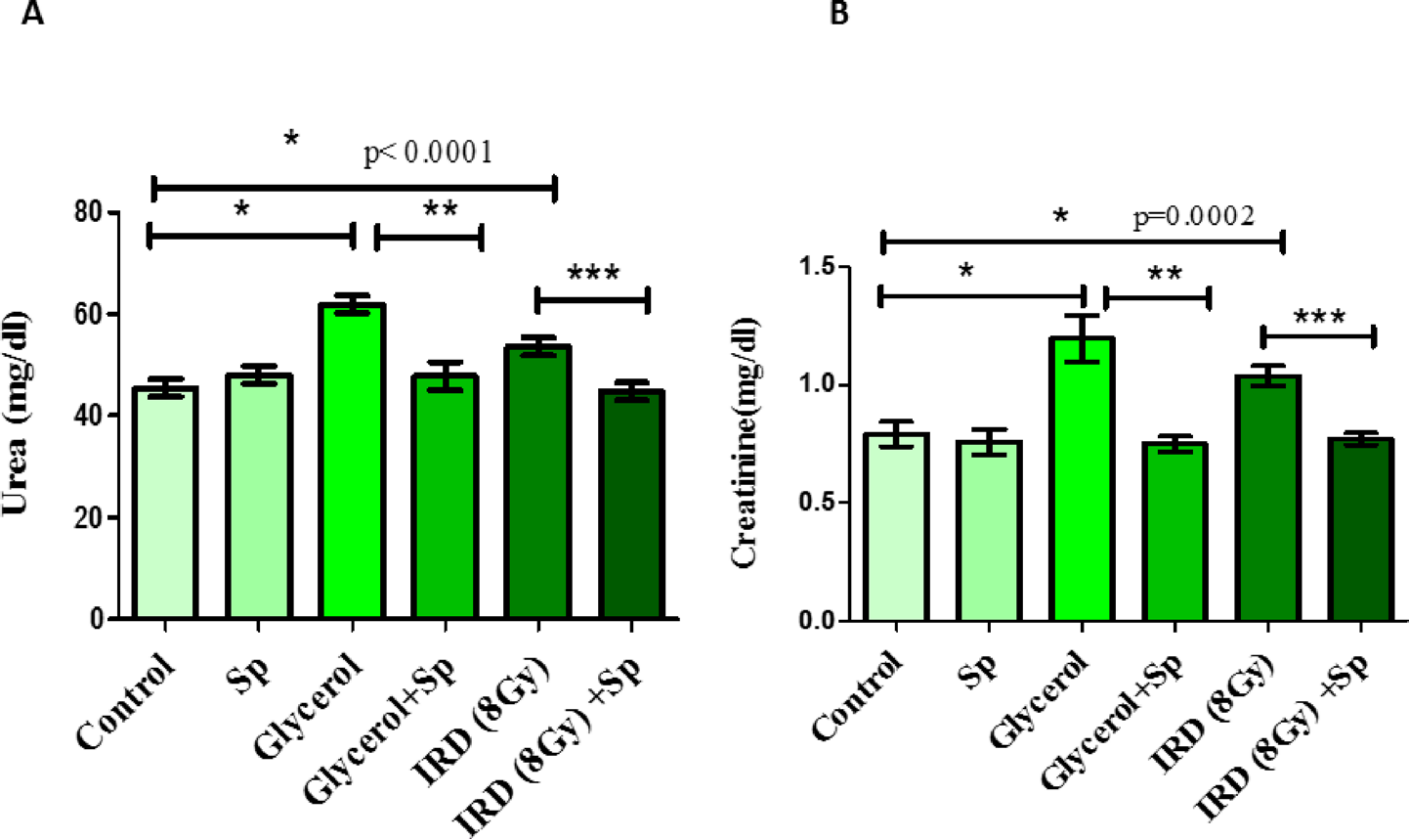
The protective role of SP administration on kidney function tests **(A)** Serum urea and **(B)** creatinine levels against glycerol or fractionated dose of gamma-rays induced renal injury in rats. Data presented as mean ± SE (*n* = 8). Statistical analysis was carried out by one-way ANOVA followed by Tukey-Kramer multiple comparisons test. * significantly different from the control group (*p* < 0.05). ** significantly different from the glycerol group (*p* < 0.05). *** significantly different from the IRD group (*p* < 0.05). Actual p-value is mentioned in the graph. SP: sodium propionate; IRD: Irradiation.

The glycerol-induced AKI group had significantly higher blood urea and creatinine levels than the control rats. Rats exposed to fractionated doses of gamma rays showed a significant increase in blood urea and creatinine levels compared to normal values (*p* < 0.05). Instead, administration of SP to rats injected by glycerol or exposed to gamma rays showed enhancements in these levels compared to positive control groups (*p* < 0.05) (Fig. 2).

### Protective role of SP on MDA and GSH oxidative stress markers against glycerol or fractionated dose of γ -rays-induced AKI in rats

**Fig. 3:**
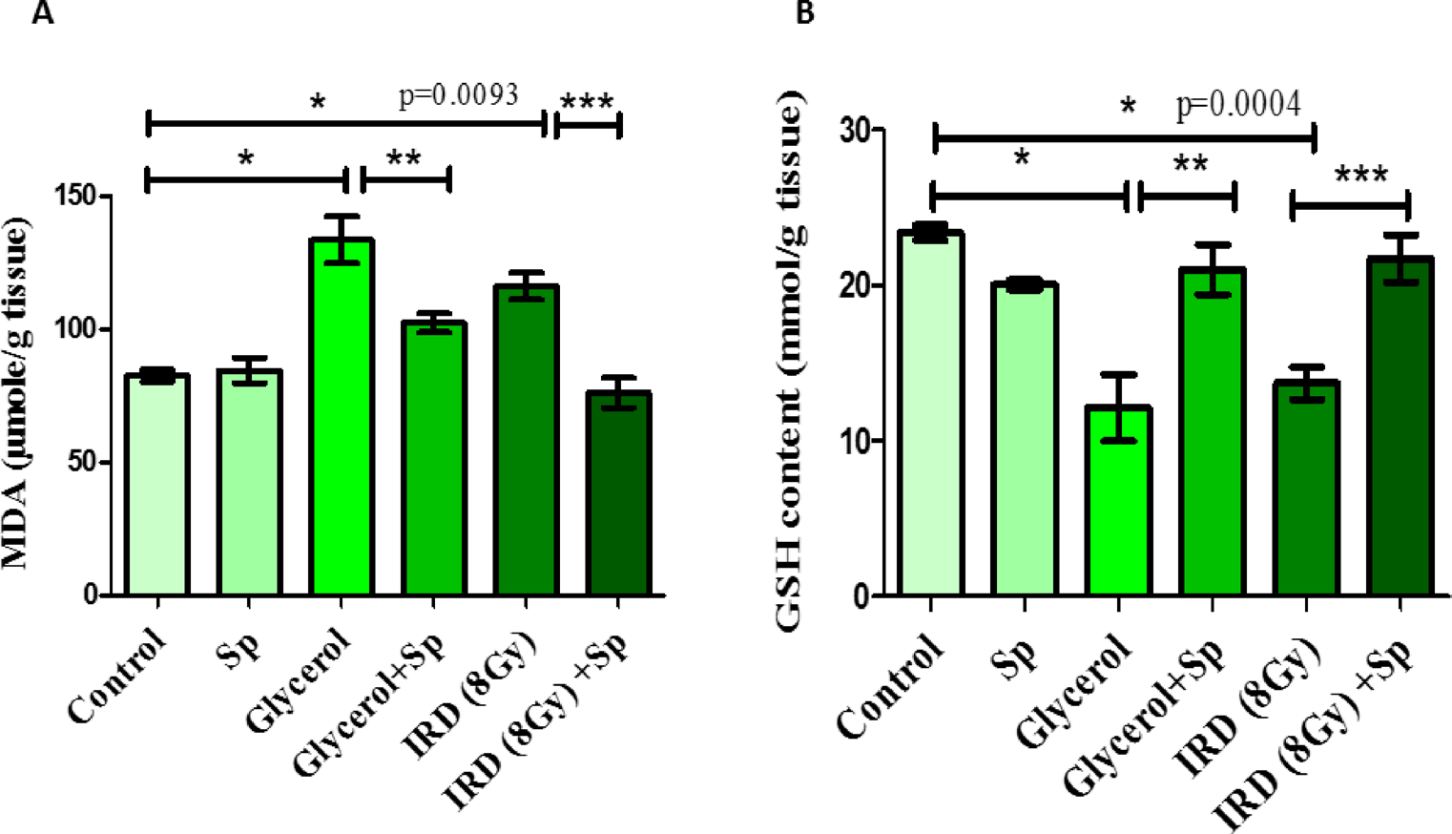
The protective role of SP administration on renal **(A)** MDA and **(B)** GSH levels against glycerol or fractionated dose of gamma-rays induced renal injury in rats. Data presented as mean ± SE (*n* = 8). Statistical analysis was carried out by one-way ANOVA followed by Tukey-Kramer multiple comparisons test. * significantly different from the control group (*p* < 0.05). ** significantly different from the glycerol group (*p* < 0.05). *** significantly different from the IRD group (*p* < 0.05). Actual p-value is mentioned in the graph. SP: sodium propionate; IRD: Irradiation.

It is hypothesized that oxidative stress plays a major role in AKI because it stimulates the expression of oxygen free radicals, which causes lipid, protein, and DNA damage. In glycerol or gamma ray-induced renal damage, renal MDA levels were much higher, while renal GSH levels were significantly lower, compared to control rats (*p* < 0.05). Compared to the positive control groups, sodium propionate reduced the levels of oxidative stress indicators (MDA and GSH) (*p* < 0.05) (Fig. 3).

### Protective role of SP on PC, MSRA and Lipofuscin oxidative stress biomarkers against glycerol or fractionated dose of γ -rays- induced AKI in rats

**Fig. 4:**
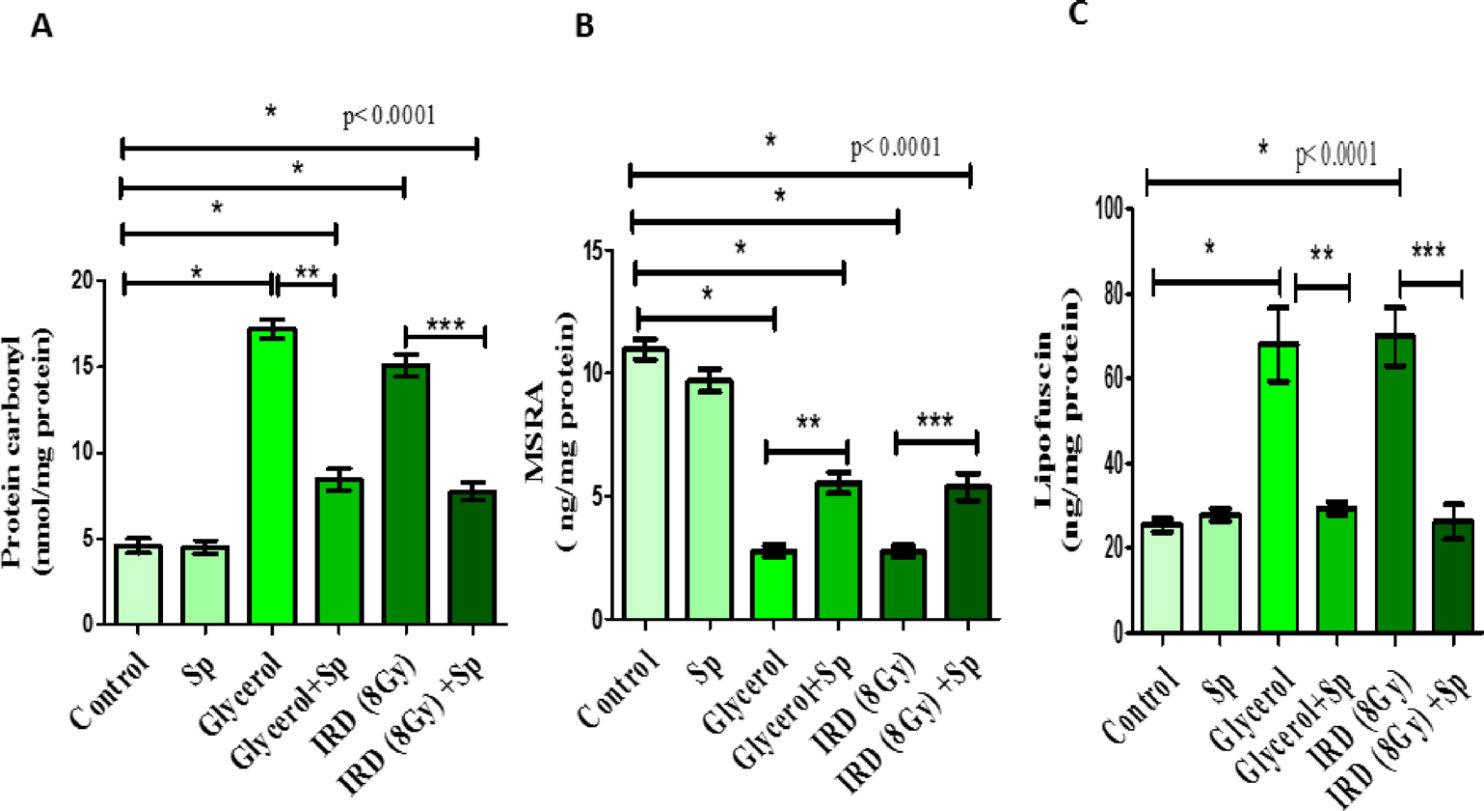
The protective role of SP administration on renal **(A)** Protein carbonyl; **(B)** Methionine sulfoxide reductase A (MSRA) and **(C)** Lipofuscin levels against glycerol or fractionated dose of gamma-rays induced renal injury in rats. Data presented as mean ± SE (*n* = 8). Statistical analysis was carried out by one-way ANOVA followed by Tukey-Kramer multiple comparisons test. * significantly different from the control group (*p* < 0.05). ** significantly different from the glycerol group (*p* < 0.05). *** significantly different from the IRD group (*p* < 0.05). Actual p-value is mentioned in the graph. SP: sodium propionate; IRD: Irradiation.

Protein carbonyl, MSRA and Lipofuscin are among the most common biomarkers of oxidative stress. Intramuscular injection of glycerol or exposure to gamma rays to rats caused notable increase in protein carbonyl and Lipofuscin, and significant decline in MSRA levels as compared to control rats (*p* < 0.05). Alternatively, oral administration of sodium propionate exerted significant improvement of these oxidative stress markers as compared to AKI groups (*p* < 0.05) (Fig. 4).

### Protective role of SP on PINK-1 and ATF 5 mitochondrial parameters against glycerol or fractionated dose of γ -rays-induced AKI in rats

**Fig. 5:**
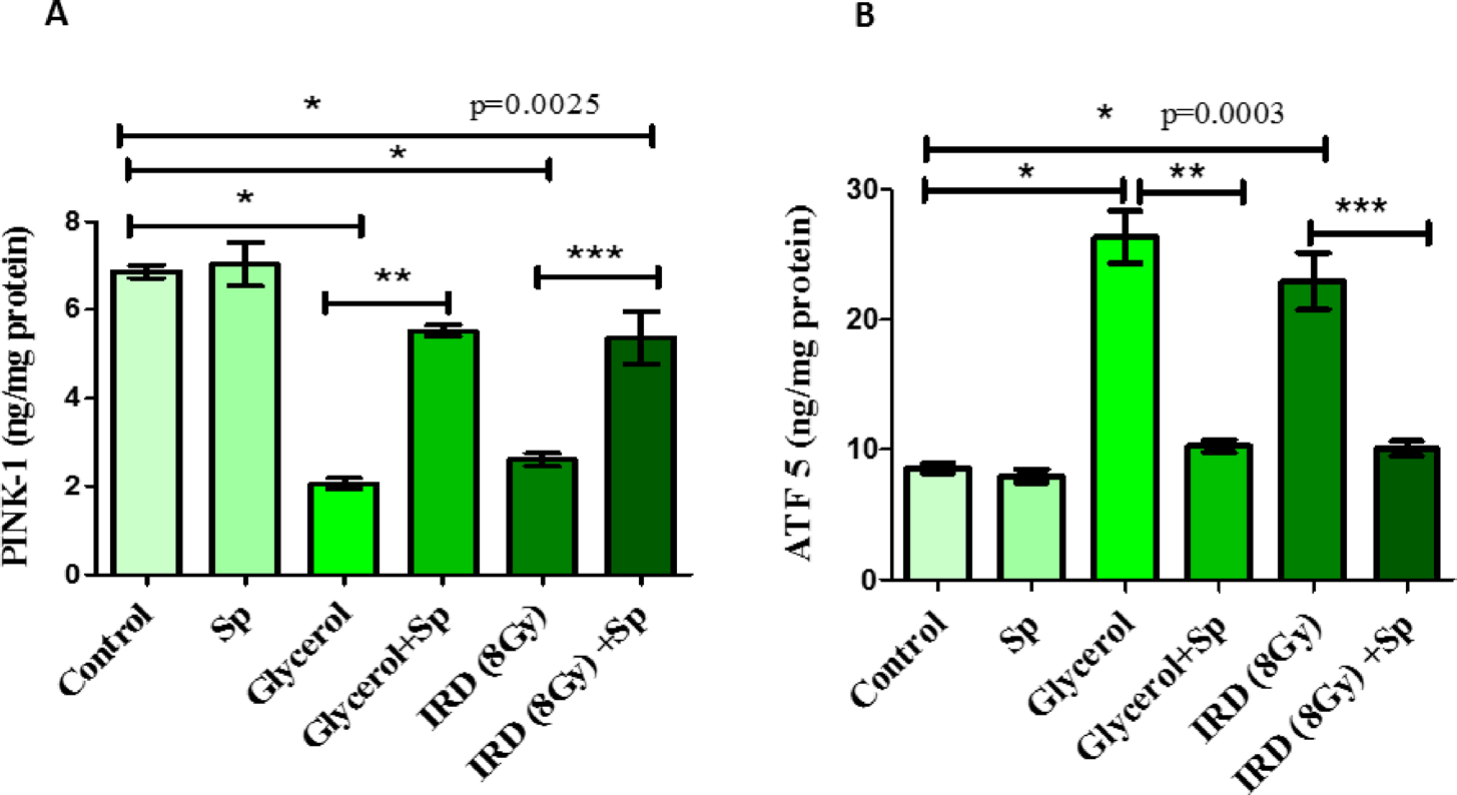
The protective role of SP administration on renal **(A)** PINK-1 and **(B)** ATF 5 levels against glycerol or fractionated dose of gamma-rays induced renal injury in rats. Data presented as mean ± SE (*n* = 8). Statistical analysis was carried out by one-way ANOVA followed by Tukey-Kramer multiple comparisons test. * significantly different from the control group (*p* < 0.05). ** significantly different from the glycerol group (*p* < 0.05). *** significantly different from the IRD group (*p* < 0.05). Actual p-value is mentioned in the graph. SP: sodium propionate; IRD: Irradiation.

AKI caused by intramuscular injection of glycerol or exposure to gamma rays to rats is associated with a notable decrease in PINK-1 levels and a substantial increase in ATF 5 levels when compared to normal rats. Whereas, treatment AKI rats with SP alleviated the levels of mitochondrial markers; PINK-1 and ATF 5 as compared to positive control values (*p* < 0.05) (Fig. 5).

### Protective role of SP on renal LC3II/LC3I ratio against glycerol or fractionated dose of gamma-rays-induced AKI in rats

**Fig. 6:**
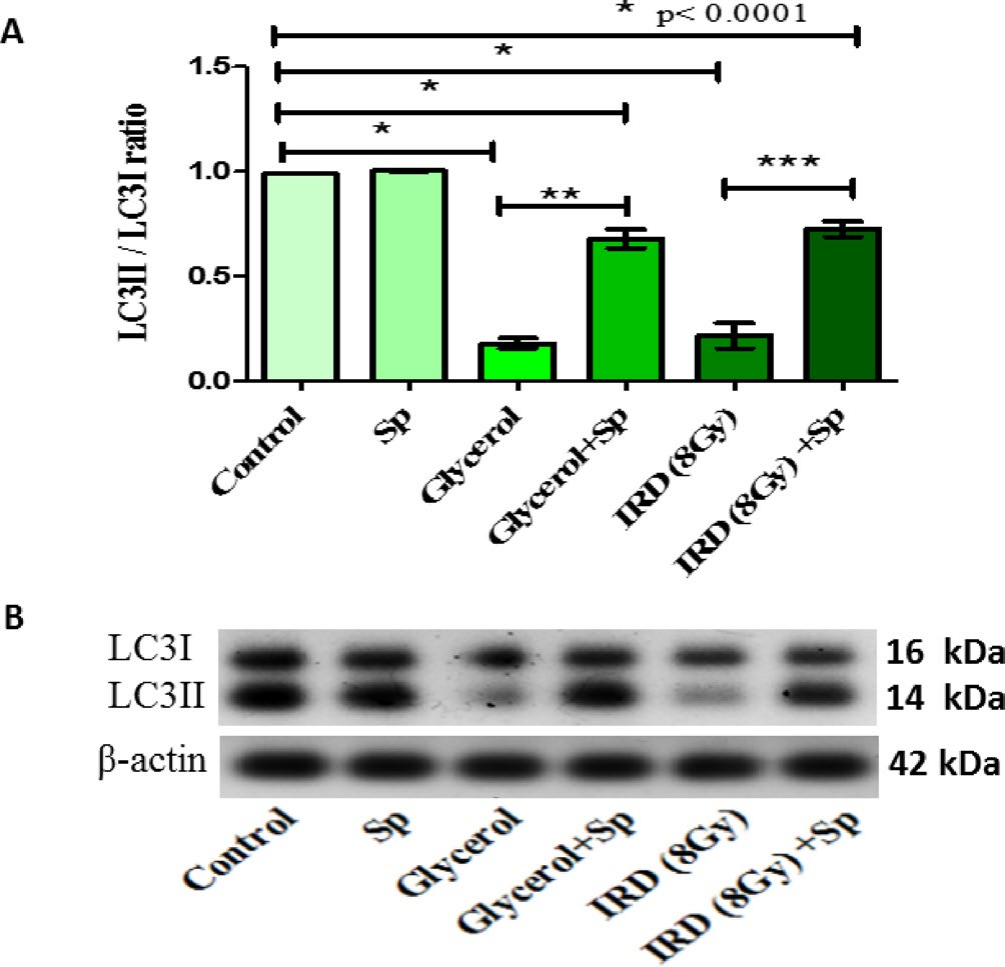
The protective role of SP administration on renal **(A)** LC3II/LC3I ratio and **(B)** protein expression and western blot analysis of LC3II/LC3I ratio against glycerol or fractionated dose of gamma-rays induced renal injury in rats. Data presented as mean ± SE (*n* = 3). Statistical analysis was carried out by one-way ANOVA followed by Tukey-Kramer multiple comparisons test. * significantly different from the control group (*p* < 0.05). ** significantly different from the glycerol group (*p* < 0.05). *** significantly different from the IRD group (*p* < 0.05). Actual p-value is mentioned in the graph. SP: sodium propionate; IRD: Irradiation. LC3 and β-actin blots were detected on the same PVDF membrane by sequential probing/stripping. A specific band was detected for β-actin at 42 kDa. Two bands: LC3-I at 16 kDa, and LC3-II at 14 kDa, respectively.

Western blot results showed that autophagy marker LC3II to I ratio is decreased in the renal tissue of AKI rats compared to control rats. While, SP significantly ameliorated the previously mentioned autophagy marker compared with those of glycerol treatment or gamma rays exposure alone **(**Fig. 6**)**.

### Protective role of SP on histopathological examinations of kidney tissues of AKI rats

Kidney tissue sections from the negative control and SP animal groups showed normal, undamaged tubular epithelial lining. No obvious inflammatory reaction or necrosis was noted. There are no visible interstitium injuries or anomalies in the interstitial compartment. Glomerular tufts appeared intact without disruption of endothelial cells.

Kidney tissue section from Glycerol injected animal group exhibited varying degrees of tubular epithelial cell damage. Renal tubules showed loss of brush border (BB), necrosis of tubular epithelial lining and intratubular albumin deposits. Glomeruli had considerable capillary tuft retraction. The inflammatory reaction was characterized by interstitial blood capillary congestion and the aggregation of a few mononuclear cells mostly lymphocytes and macrophages. In contrast, SP significantly improved histological markers in glycerol-treated group (Group IV) compared with glycerol per se, with mild swelling of the tubular epithelial lining, loss of BB Glomeruli showed hypercellularity of capillary tufts and narrowing of Bowman’s space. There was no inflammation or bleeding.

**Fig. 7:**
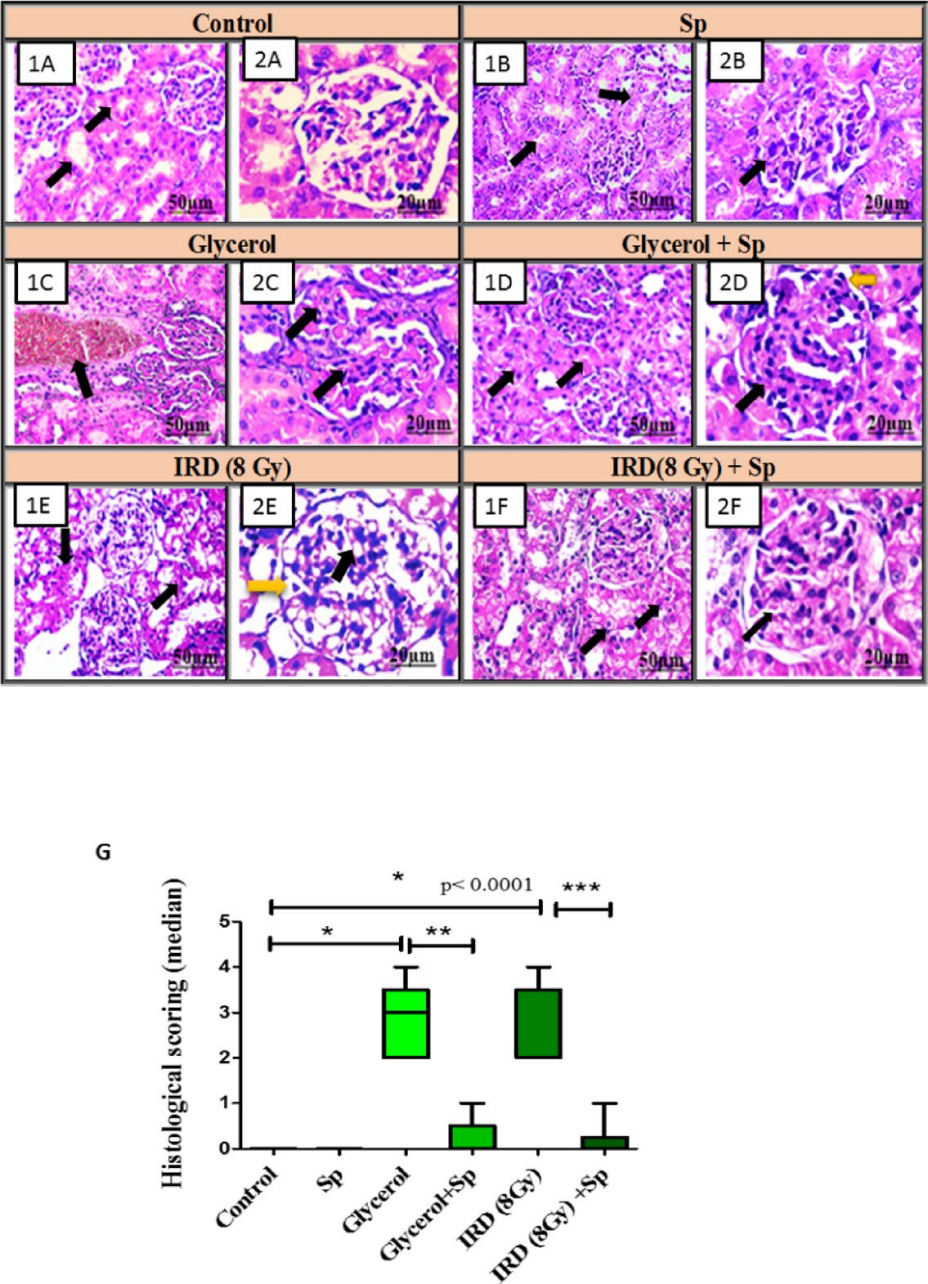
**(A)** Photomicrograph of kidney tissue of normal rats section showing: **(1 A)** normal intact tubular epithelial lining **black arrow** (x200) scale bars: 50 μm **(2 A)** normal histological structure of glomerulous **black arrow** scale bars: 20 μm (H&Ex400). **(B)** Photomicrograph of kidney tissue of SP-treated rats section showing: **(1B)** normal intact tubular epithelial lining **black arrow** scale bars: 50 μm (x200) **(2B)** normal histological structure of glomerulous **black arrow** scale bars: 20 μm (H&Ex400). **(C)** Photomicrograph of kidney tissue of glycerol group section showing: **(1 C)** congestion of interstitial blood capillaries µm **black arrow** scale bars: 50 μm (x200) **(2 C)** retraction of glomerular tufts scale bars: 20 μm **black arrow** (H&Ex400). **(D)** Photomicrograph of kidney tissue of glycerol + SP group section showing: **(1D)** swelling of tubular epithelial lining scale bars: 50 μm **black arrow** (x200) **(2D)** hypercellularity of capillary tufts **black arrow** and narrowing of Bowman’s space **yellow arrow** scale bars: 20 μm (H&Ex400). **(E)** Photomicrograph of kidney tissue of gamma rays (8 Gy) group section showing: **(1E)** necrosis of tubular epithelial lining **black arrow** scale bars: 50 μm (x200) **(2E)** dilatation of capillary tufts **black arrow** and narrowing of Bowman’s space **yellow arrow** scale bars: 20 μm (H&Ex400). **(F)** Photomicrograph of kidney tissue gamma rays (8 Gy) + SP group section showing: **(1 F)** mild swelling of tubular epithelial lining **black arrow** scale bars: 50 μm (x200) **(2 F)** intact Bowman capsule **black arrow** scale bars: 20 μm (H&Ex400). **(G)** Histological scoring of renal damage. Each value represents the median (interquartile ranges) of three experiments. * significantly different from the control group (*p* < 0.05). ** significantly different from the glycerol group (*p* < 0.05). *** significantly different from the IRD group (*p* < 0.05) using Kruskal–Wallis test followed by post-hoc test (Dunn’s test). Actual p-value is mentioned in the graph. SP: sodium propionate; IRD: Irradiation.

Kidney tissue sections from rats exposed to 8 Gy gamma-radiation revealed tubular epithelial lining necrosis with intratubular albuminus deposits. Glomeruli demonstrated significant dilatation of capillary tufts, constriction of Bowman’s space. No inflammation or hemorrhages were seen. On the other hand, kidney tissue sections from (8 Gy + SP) animals showed significant comparative improvements compared to the previous group, with modest swelling of the tubular epithelial lining and loss of Brush Border (BB). Glomeruli displayed an intact Bowman capsule without retraction of capillary tufts, as well as the absence of inflammation, blood capillary congestion, hemorrhages, and tubular necrosis (Fig. 7) and Table 2.

**Table 2:** Histopathological scoring of renal damage.

Score	Vacuolization	Cortical damage	Necrosis
Control	0	0	0
SP	0	0	0
Glycerol	3	4	3
Glycerol + SP	2	1	0
IRD (8 Gy)	4	3	3
IRD (8 Gy) + SP	1	1	1

While both models induced significant AKI, we observed that glycerol primarily caused severe cortical acute tubular necrosis and more pronounced myoglobinuric tubular cast formation related oxidative stress. In contrast, radiation damage was more closely associated with DNA damage and exhibited higher levels of glomerular hemodynamic disruption and vascular congestion.

### Protective role of SP on P62 immunohistochemical protein expression against glycerol or fractionated dose of gamma-rays induced AKI in rats

**Fig. 8:**
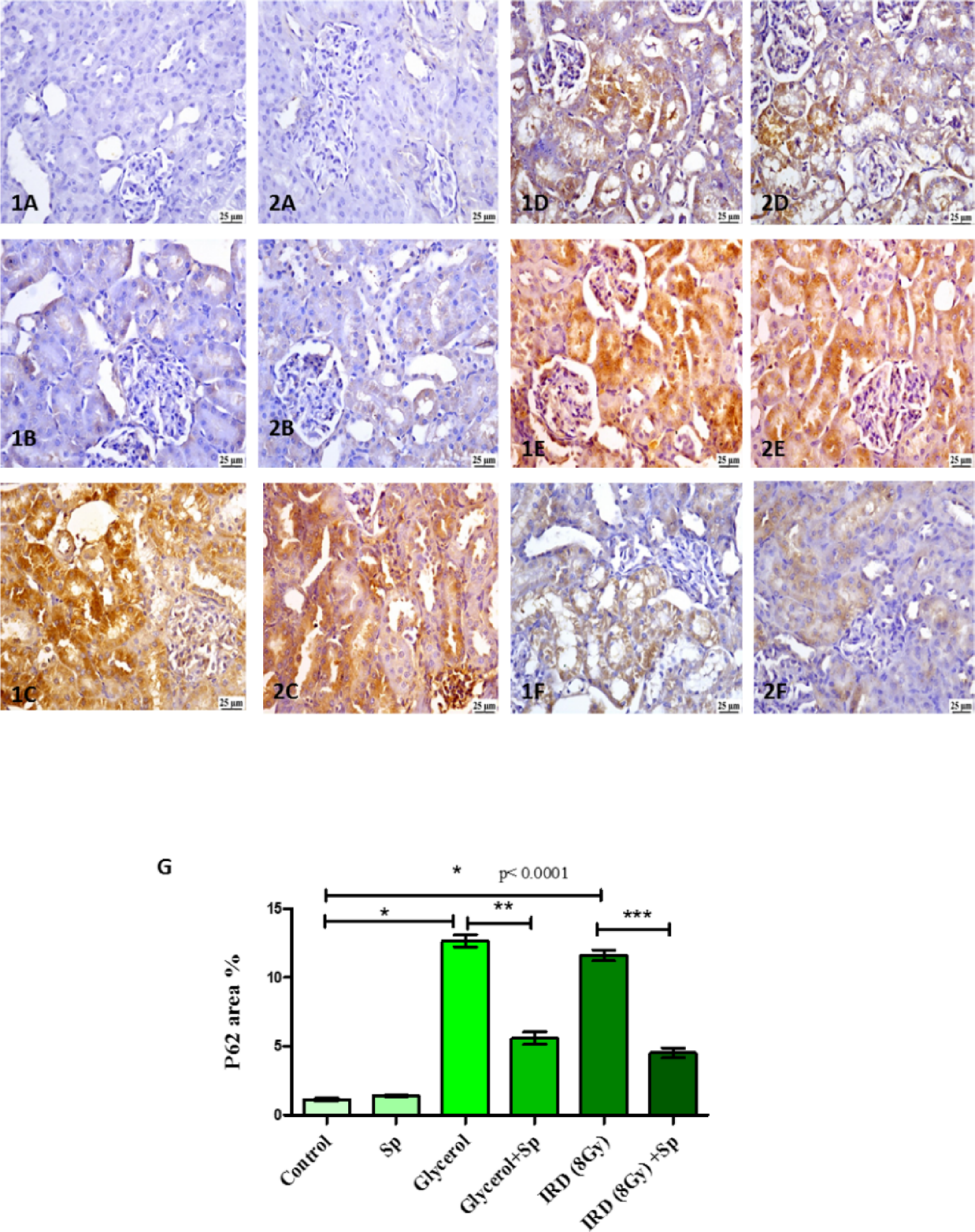
**(1 A**,** 2 A)** Photomicrograph of kidney **control** group showing weak P62 expression (Immune staining). **(1B**,** 2B)** Photomicrograph of Kidney **SP** group showing lower P62 expression (Immune staining). (**1 C**,** 2 C**) Photomicrograph of Kidney **Glycerol** group showing higher P62 expression (Immune staining). (**1D**,**2D**) Photomicrograph of Kidney **Glycerol + SP** group showing moderate P62 expression (Immune staining). (**1E**,** 2E**) Photomicrograph of Kidney **radiation** group showing higher P62 expression (Immune staining). (**1 F**,** 2 F**) Photomicrograph of Kidney **radiation + SP** group showing moderate P62 expression (Immune staining). (**G**) Chart represents P62 expression as mean area percent of positive staining. Data are presented as mean ± SE. Significant difference is considered at *P* < 0.05. Actual p-value is mentioned in the graph. SP: sodium propionate; IRD: Irradiation.

Examination of the renal parenchyma of different groups revealed lower P62 expression in control and SP groups. Meanwhile, there was a considerable increase in the glycerol group compared to the other groups except gamma radiation group. A significant decrease in P62 expression was determined in gamma radiation (8 Gy) + SP group in comparison with gamma radiation group (Fig. 8).

## Discussion

This study demonstrated that SP exerts a renoprotective effect against AKI induced by glycerol or fractionated gamma radiation in rats. SP significantly ameliorated kidney function, as evidenced by reduced serum urea and creatinine levels. Mechanistically, SP administration significantly decreased oxidative stress markers (MDA, lipofuscin, and protein carbonyl) and restored antioxidant capacity markers (GSH and MSRA). Furthermore, SP modulated mitochondrial quality control by normalizing of mitophagy-related biomarkers (PINK-1 and LC3II/LC3I ratio) and decreasing the mitochondrial unfolded protein response (UPR^mt^) transcription factor ATF5 and autophagy adapter p62 expression. These biochemical and molecular improvements were corroborated by a significant amelioration of renal histopathological changes.

Excessive glycerol metabolism, especially in the presence of inadequate antioxidant defenses, can cause oxidative stress, which is defined as an imbalance between ROS formation and the cell’s ability to detoxify reactive intermediates. This oxidative stress can harm cellular components, such as lipids, proteins, and DNA, and disrupt mitochondrial function, potentially leading to apoptosis or other forms of cell death^17,56^. Our findings confirmed this pathology, as both glycerol and gamma irradiation significantly reduced the antioxidant markers GSH and MSRA while markedly elevating lipid peroxidation (MDA), protein damage (protein carbonyl/PC), and cellular debris (lipofuscin) levels in the renal tissue.

Our results are consistent with earlier research that has linked oxidative stress damage and renal tubular apoptosis in glycerol-induced renal impairment^19,57^. Moreover, these results are in agreement with previous studies linking oxidative stress to nephrotoxicity induced by chemotherapeutic agents such as cisplatin and methotrexate. In cisplatin-induced AKI, hesperidin conferred dose-dependent renoprotection through modulation of autophagy markers Beclin‑1 and LC3‑II^58^. Similarly, vanillic acid has been shown to attenuate methotrexate induced nephrotoxicity via its anti-apoptotic, antioxidant, and anti-inflammatory properties^59,60^. Together, these findings reinforce the central role of oxidative stress in diverse models of renal injury and highlight the therapeutic potential of antioxidant interventions.

Methionine sulfoxide reductases (MSRs) are enzymes that play a crucial role in repairing oxidatively modified proteins by reducing methionine residues back to their original form^61^. MSRA included in this study to reflect its central role in maintaining protein integrity under oxidative stress. Significant declines in MSR may be linked to high levels of oxidative stress, which can directly destroy proteins such as MSR^62^. This damage can result in diminished enzyme activity or even complete loss of function, effectively decreasing the quantities of active MSRA^63^. By highlighting MSRA, this work emphasizes the vulnerability of protein repair systems during AKI and positions MSRA as a potential therapeutic target for mitigating oxidative damage and preserving mitochondrial function.

Elevated oxidative stress also leads to the carbonylation of amino acid side chains in proteins, resulting in increased PC formation. PC is a widely accepted indicator of severe oxidative damage, leading to impaired enzymatic activity and disrupted cellular signaling^64^. Additionally, both injury models deplete endogenous antioxidants like glutathione, promoting lipid peroxidation that yields reactive aldehydes such as MDA, which further contribute to protein carbonylation^65,66^.The observed increase in protein carbonyl levels in glycerol-treated rats is consistent with findings of *Kassab et al.*^67^, who demonstrated that glycerol (50%, 10 ml/kg, i.m.) for induction of AKI, indicated decline in reduced glutathione (GSH) levels accompanied by an elevation in MDA and nitric oxide (NO) levels. Additionally, glycerol administration significantly down-regulated gene expression levels of nuclear factor erythroid 2-related factor 2 (Nrf2) and hemeoxygenase-1 (HO1)^68^. Furthermore, γ-irradiation can directly induce DNA damage and genomic instability, particularly in rapidly dividing cells, leading to apoptosis or necrosis in the renal epithelium^69–73^.

Both glycerol and gamma radiation can produce free radicals that harm biological components such as lipids, proteins, and DNA. This oxidative damage may contribute to the buildup of lipofuscin. Additionally, oxidative stress can hamper autophagic clearance systems, resulting in the accumulation of undegraded cellular debris that contributes to lipofuscin development^74,75^. Although lipofuscin is traditionally regarded as a marker of cumulative and age-related oxidative damage, its estimation in the present AKI model is justified by the intensity of oxidative stress and impaired proteostasis that accompany acute insults^76^.

AKI is characterized by rapid depletion of antioxidants, excessive lipid peroxidation, and mitochondrial dysfunction, all of which accelerate the formation of lipofuscin granules. Importantly, lipofuscin accumulation does not require prolonged time frames alone; rather, it reflects the inability of lysosomal systems to degrade oxidized macromolecules under conditions of overwhelming stress^77^. Thus, even in acute settings, lipofuscin serves as an integrative marker that complements transient indicators such as malondialdehyde or protein carbonyls by capturing the persistence of oxidative injury and lysosomal overload^78^. Lipofuscin estimation therefore, strengthens the mechanistic interpretation of AKI pathology, highlighting that acute oxidative stress can initiate cellular changes typically associated with chronic injury, and underscoring its potential utility in evaluating therapeutic interventions aimed at restoring proteostasis.

Sodium propionate administration along with glycerol or gamma irradiation significantly decreased oxidative stress related biomarkers. As a short-chain fatty acid (SCFA) salt, sodium propionate antioxidant activity is attributed to its ability to scavenge reactive oxygen species (ROS) and increase the expression of endogenous antioxidant enzymes, such as superoxide dismutase (SOD) and glutathione peroxidase (GPx), thereby reducing oxidative stress and protecting cells from oxidative damage^48^.Our results agree with a previous work, reported that SP increased the activity of antioxidant enzymes such superoxide dismutase (SOD), catalase (CAT), and glutathione peroxidase (GPx)^79^. Nrf2 translocates to the nucleus and stimulates the development of antioxidant proteins, reinforcing redox homeostasis and protecting renal tissue^80^.

Our study indicates that protective mechanism of SP extends beyond its antioxidant activity to include modulation of mitophagy, a specialized form of autophagy responsible for the selective clearance of damaged mitochondria and preservation of cellular homeostasis^81^. Mitophagy is tightly regulated by key signaling pathways, including the PINK1-Parkin axis and receptor-mediated mechanisms involving proteins such as Bcl-2 interacting protein 3 (BNIP3) and FUN14 Domain Containing 1(FUNDC1)^82^. Oxidative stress is a major driver of mitochondrial damage, and impaired mitophagy can establish a vicious cycle that perpetuates oxidative stress and leads to cellular pathology^83–85^.

In both injury models, glycerol or gamma radiation exposure led to a significant decrease in the mitophagy marker PINK-1 and a diminished LC3II/LC3I ratio, indicating impaired mitophagy flux. Consistently, immunohistochemistry revealed marked accumulation of the autophagy adaptor protein p62, which is normally degraded during active autophagy. Elevated p62 levels therefore serve as a robust indicator of compromised autophagic flux under severe oxidative stress. The results are consistent with those obtained by *Habieb et al.*^86^, who attributed elevated levels of autophagic p62 protein to decreased autophagy induction as confirmed by lower LC3II to LC3I ratio.

The decline in PINK-1 expression is attributed to the distinct mechanisms of glycerol-induced osmotic stress can impair PINK-1 import and stabilization on the outer mitochondrial membrane^87^, while gamma radiation-induced ROS and DNA damage can degrade mitochondrial function and lower PINK-1 stability^88^. Mitophagic deficiency through PINK-1 loss has been shown to enhance tubular cell apoptosis and tissue damage, while increasing mitochondrial dysfunction, mitochondrial ROS formation, DNA oxidative injury, and inflammation in cisplatin-induced AKI and renal ischemia-reperfusion models^89,90^. This finding, however, contrasts with a prior study that reported radiation-induced mitophagy as a protective response against renal tissue damage^91^.

Furthermore, glycerol and γ-irradiation caused a notable increase in the renal expression of activating transcription factor 5 (ATF5). This elevation is likely a result of the overwhelming oxidative stress leading to mitochondrial destruction and the release of misfolded mitochondrial proteins into the cytosol. While this initially activates the mitochondrial unfolded protein response (UPR^mt^), sustained oxidative stress overwhelms the cell adaptive capacity, culminating in ATF5 upregulation, which is crucial for mitochondrial homeostasis^92^. Our results agree with *Das et al.*^93^, who indicated that irradiated cells showed increased phosphorylation of GCN2, PERK, and eIF2α, which may lead to up-regulation of ATF4 and CHOP, controlling autophagy and integrated stress response. Furthermore, ionizing radiation increases the accumulation of mitochondrial chaperones (HSP60/HSP10) and ATF5, a major molecule involved in mitochondrial stress, which leads to the activation of adaptive mechanisms like cytoprotective autophagy and molecules responsible for mitochondrial biogenesis and protein quality control in order to replenish the mitochondrial pool and maintain cellular homeostasis^94^.

SP treatment effectively counteracted these changes, significantly restoring PINK-1 and LC3II/LC3I levels while alleviating the ATF5 and p62 upregulation. This significant autophagic inducing effect may be a direct result of SP ability to reduce oxidative stress, thereby restoring PINK-1 stability and promoting a protective autophagic flux. Re-establishing autophagy helps remove damaged organelles and acts as an adaptive strategy to prevent mitochondria-mediated apoptotic cell death, thereby protecting the kidney cells from injury and promoting repair^95,96^.

The kidney high vascularization and metabolic activity make it highly vulnerable to oxidative stress^97^. Consistent with this, both glycerol and γ-irradiation caused significant histological alterations, including tubular necrosis, epithelial cell death, vacuolization, and inflammation. Specifically, glycerol induced injury was characterized by tubular cell death, vacuolization (possibly from osmotic stress or lipid accumulation), and inflammation in the renal cortex. In contrast, γ-irradiation caused more widespread tissue damage, evidenced by tubular epithelial cell death (necrosis/apoptosis) and potential fibrosis due to extracellular matrix deposition^98,99^. Crucially, SP administration significantly alleviated the histological damage in the renal tissue caused by both glycerol and γ-irradiation. The improvements in interstitial and tubular scores were particularly notable, as SP significantly reduced inflammation and necrosis in the glycerol group and completely prevented necrosis in the radiation group. These structural improvements directly correlate with the observed amelioration of biochemical and oxidative stress parameters, supporting a generalized protective role for SP against both AKI etiologies.

## Conclusion

To the best of our knowledge, this is the first study to compare the renoprotective effect of SP against AKI induced by two distinct etiologies: glycerol induced rhabdomyolysis and γ-irradiation. Our comprehensive findings highlight a common mechanism of action: SP antioxidant and mitophagy restoring properties effectively combat the severe oxidative stress and mitochondrial dysfunction that are shared pathological features of both AKI models. This study provides a strong mechanistic rationale for exploring SP as a promising therapeutic agent for a broader spectrum of AKI types. While our study focused on renal tissue, systemic irradiation suggests potential protective effects in other organs (e.g., lung, heart), warranting further investigation. Pre-clinical and clinical studies are essential to fully elucidate SP regulatory mechanisms and therapeutic potential.

## Supplementary Information

Below is the link to the electronic supplementary material.

Supplementary Material 1

Supplementary Material 2

Supplementary Material 3

Supplementary Material 4

Supplementary Material 5

## Data Availability

The datasets used and/or analyzed in the current investigation are available from the corresponding author upon reasonable request.
