# Supplementary figures and images for: Protective role of sodium propionate against glycerol or fractionated doses of gamma rays-induced acute kidney injury via ATF5-induced mitophagy in rats
Source: Sci Rep. 2026 Apr 12;16:12073. doi: 10.1038/s41598-026-46553-3 (PMC13077041; doi:10.1038/s41598-026-46553-3)

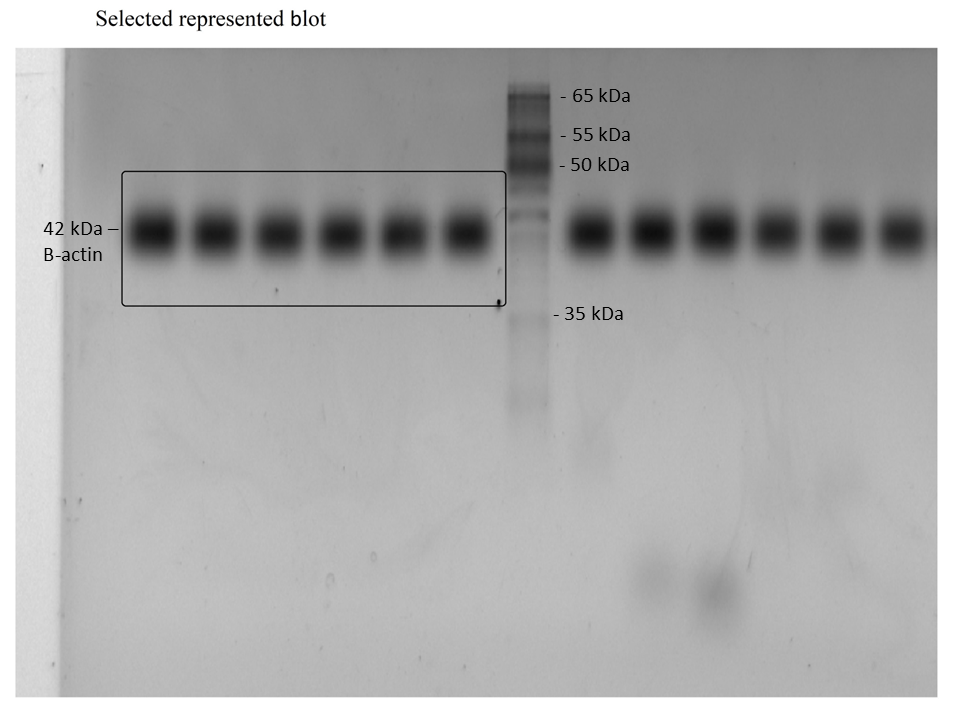

Supplement: Supplementary file 1 — Supplementary Material 1 [file 41598_2026_46553_MOESM1_ESM.tif]

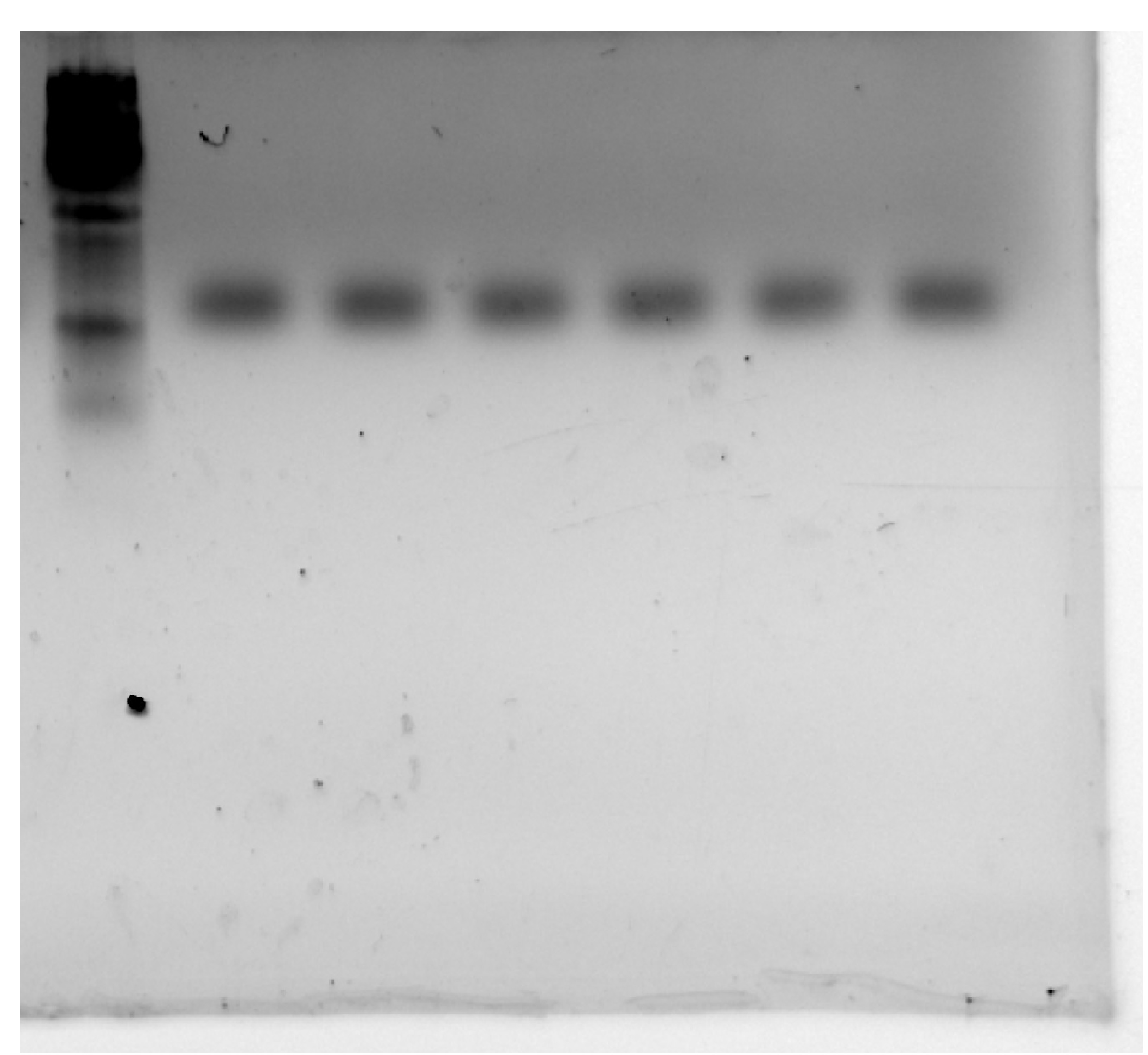

Supplement: Supplementary file 2 — Supplementary Material 2 [file 41598_2026_46553_MOESM2_ESM.jpg]

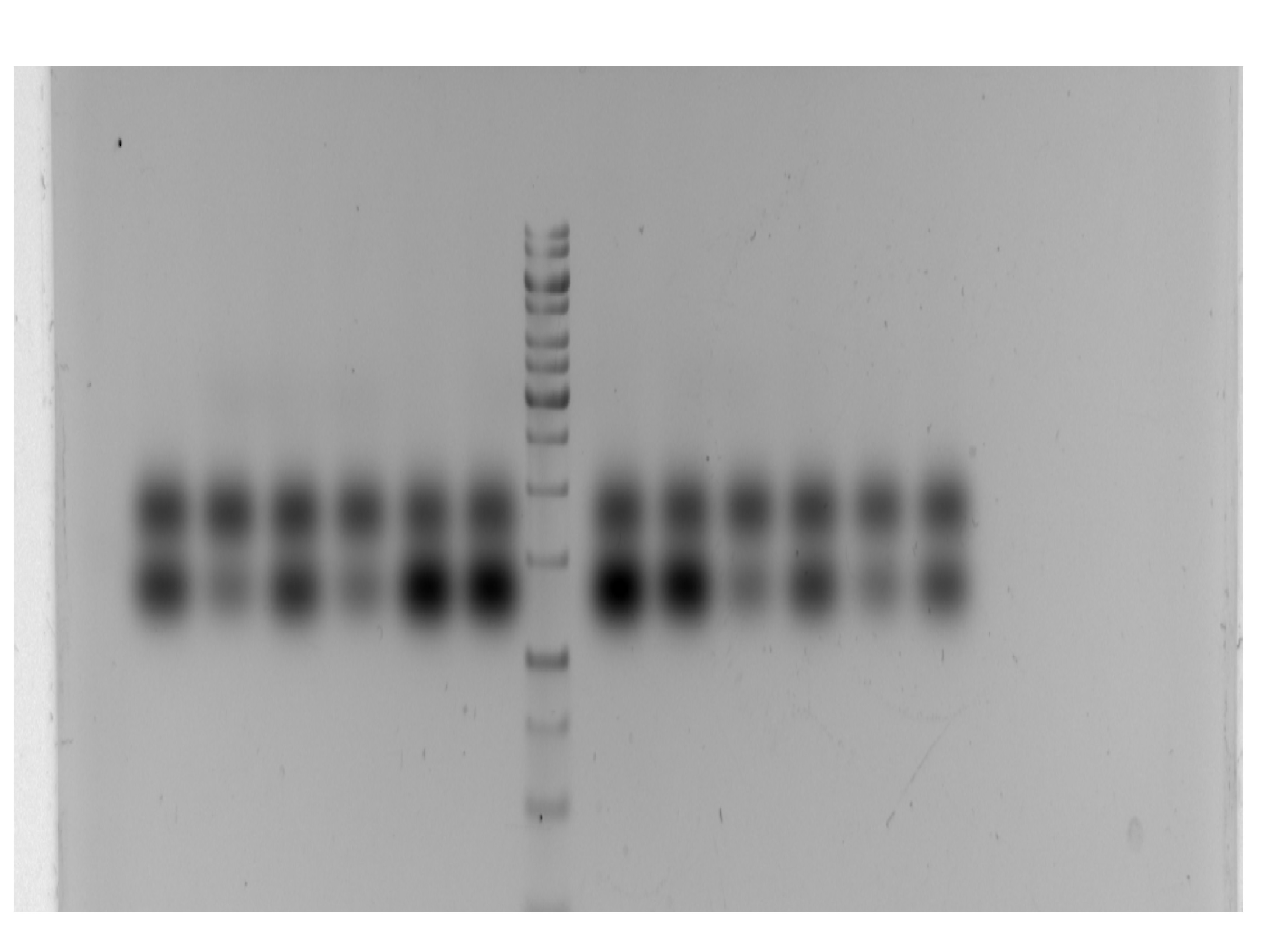

Supplement: Supplementary file 3 — Supplementary Material 3 [file 41598_2026_46553_MOESM3_ESM.jpg]

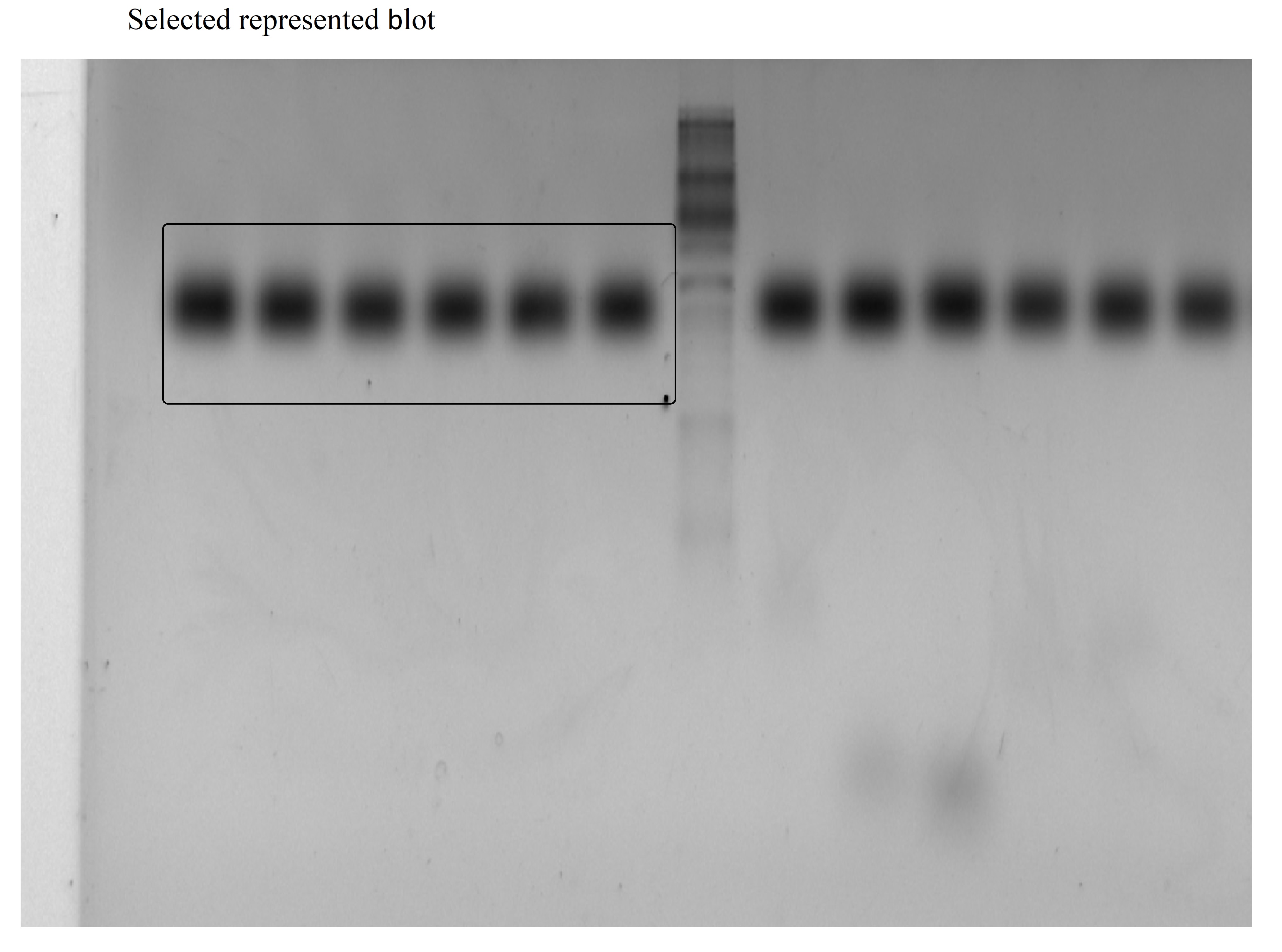

Supplement: Supplementary file 4 — Supplementary Material 4 [file 41598_2026_46553_MOESM4_ESM.jpg]

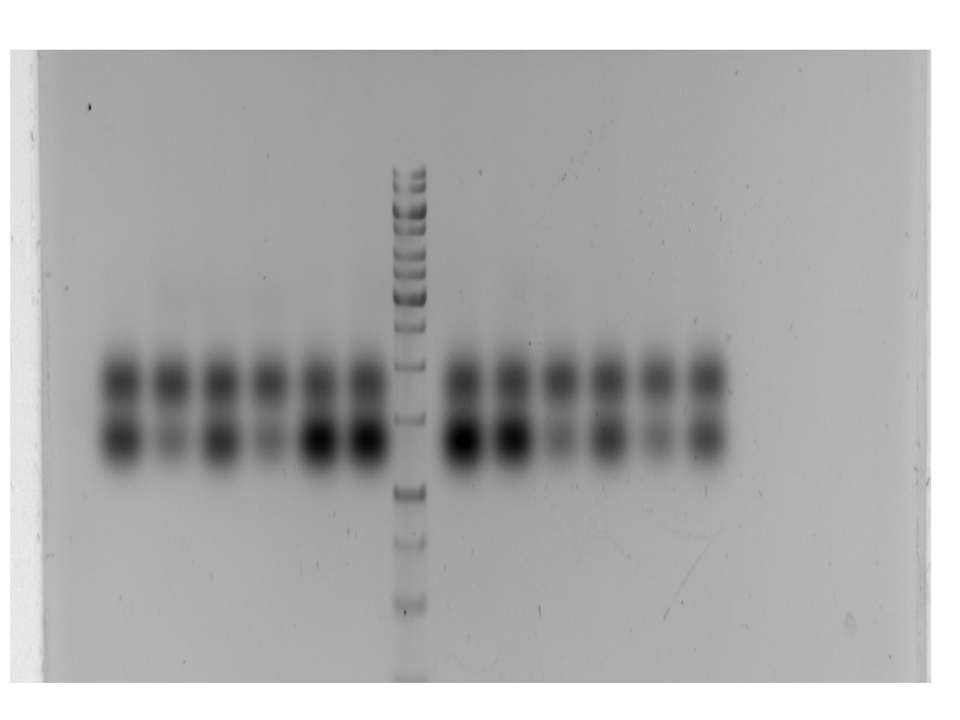

Supplement: Supplementary file 5 — Supplementary Material 5 [file 41598_2026_46553_MOESM5_ESM.tif]
